# Matcha Tea Powder’s Antidepressant-like Effect through the Activation of the Dopaminergic System in Mice Is Dependent on Social Isolation Stress

**DOI:** 10.3390/nu15030581

**Published:** 2023-01-22

**Authors:** Yuki Kurauchi, Yuki Ohta, Keigo Matsuda, Wakana Sanematsu, Hari Prasad Devkota, Takahiro Seki, Hiroshi Katsuki

**Affiliations:** 1 Department of Chemico-Pharmacological Sciences, Graduate School of Pharmaceutical Sciences, Kumamoto University, Kumamoto 862-0973, Japan; 2 Department of Instrumental Analysis, Graduate School of Pharmaceutical Sciences, Kumamoto University, Kumamoto 862-0973, Japan; 3 Headquarters for Admissions and Education, Kumamoto University, Kumamoto 860-8555, Japan; 4 Department of Pharmacological Sciences, Faculty of Pharmaceutical Sciences, Himeji Dokkyo University, Himeji 670-8523, Japan

**Keywords:** depression, dopaminergic circuit, Matcha, C57BL/6J, BALB/c

## Abstract

Matcha tea powder is believed to have various physiological benefits; however, its detailed mechanism of action has been poorly understood. Here, we investigated whether the mental state of mice, due to social isolation stress, affects the antidepressant-like effect of Matcha tea powder by using the tail suspension test. Oral administration of Matcha tea powder reduced the duration of immobility in the stress-susceptible C57BL/6J strain, but not in BALB/c strain. In C57BL/6J mice, SCH23390, a dopamine D1 receptor blocker, prevented Matcha tea powder from exerting its antidepressant-like effect. Matcha tea powder also increased the number of c-Fos-positive cells in the prefrontal cortex (PFC) region and the nucleus accumbens (NAc) region in C57BL/6J mice, but not in BALB/c mice. In contrast, Matcha tea powder did not change the number of c-Fos-positive cells in the ventral tegmental area (VTA) region. Notably, C57BL/6J mice with a shorter immobility time had a higher number of c-Fos-positive cells in the PFC, NAc, and VTA regions. However, no such correlation was observed in the stress-tolerant BALB/c mice. These results suggest that Matcha tea powder exerts an antidepressant-like effect through the activation of the dopaminergic system including the PFC-NAc-VTA circuit and that mental states are important factors affecting the physiological benefits of Matcha tea powder.

## 1. Introduction

Depression is the most-prevalent psychiatric disorder in the world, and the number of patients with depression is continuously increasing worldwide [1]. Although there are individual differences in the onset of depression, its pathogenesis is thought to involve a reduction in dopaminergic function in the brain. Postmortem brain analysis and positron emission tomography (PET) radioligand studies in humans have shown that dopamine D1 receptor expression and function are significantly reduced in the patients with major depressive disorder [2]. It has also been reported that the activation of dopamine D1 receptors improves symptoms in a mouse model of depression [3]. Various antidepressants have been developed, including selective serotonin reuptake inhibitors (SSRIs), serotonin and norepinephrine reuptake inhibitors (SNRIs), and tricyclic antidepressants, which increase the monoamine levels in the brain; however, the risk of side effects is high, and treatment-resistant depression has recently become a social problem. However, the mechanisms by which individual differences exist in the onset and treatment efficacy of depression are poorly understood.

In the search for safer natural alternatives to these medicines, various natural products and plant extracts have been studied for their potential antidepressant properties [4,5,6]. Matcha is a finely ground powder made from the leaves of a specially cultivated tea plant (*Camellia sinensis*) and is traditionally consumed as tea by suspending it in hot water. In recent years, Matcha has gained worldwide attention for its use in dietary supplements and flavoring ingredient in snacks [7]. The leaves of *Camellia sinensis* are rich in polyphenols including catechins, caffeine, and l-theanine, and these compounds have been considered to be effectors of mood and mental performance in humans and mice [8,9,10,11,12,13,14,15,16,17,18]. We previously reported that Matcha tea powder improved anxiety-like behavior in healthy C57BL/6J mice and that the activation of dopamine D1 receptor signaling was involved in the mechanism [19]. Therefore, Matcha tea powder is expected to have the potential to activate dopaminergic functioning to improve depression. However, the antidepressant-like effects of Matcha tea powder itself have not been demonstrated in animal models. Furthermore, no studies have focused on the activation states of dopaminergic neuronal circuits with respect to the antidepressant-like effects of Matcha tea powder.

In this study, we investigated the antidepressant-like effects of Matcha tea powder using the tail suspension test (TST) in socially isolated male C57BL/6J and BALB/c mice. Although C57BL/6J and BALB/c mice have been commonly used in experiments, no studies have directly compared the association between different mental status and the antidepressant-like effects of Matcha tea powder. Furthermore, we focused on the dopaminergic systems in the brain and investigated the contribution of the dopamine D1 receptors to the antidepressant-like effects of Matcha tea powder itself.

## 2. Materials and Methods

### 2.1. Animals

All procedures were approved by the Kumamoto University Ethics Committee on Animal Experiments (approval number: A28-048), and the animals were treated in accordance with the Guiding Principles for the Care and Use of Laboratory Animals. Male C57BL/6J and BALB/c mice at 7 weeks of age weighing 20.8–25.5 g (C57BL/6J mice) and 20.1–25.3 g (BALB/c mice) were provided by Nihon SLC (Shizuoka, Japan). All animals were maintained individually for 1 week at a constant ambient temperature (22 ± 1 °C) under a 12 h light/dark cycle (light from 8:00 to 20:00), with food and water available ad libitum. The total number of mice used in the experiments was 190 (C57BL/6J mice; *n* = 95, BALB/c mice; *n* = 95).

### 2.2. Preparation of Matcha

The Matcha tea powder used in this study was provided by AIYA Co. Ltd. (Nishio, Aichi, Japan) and was prepared using only the fresh new leaves of *Camelia sinensis*, harvested in Aichi, Japan.

### 2.3. Dosage Information

The dosage of Matcha tea powder was based on our previous study [19]. Matcha tea powder was suspended in sterilized water at 1, 3, and 10 mg/mL and orally administered using a feeding needle (CLEA Japan, Inc., Tokyo, Japan) at a dose of 10, 30, and 100 mg/kg, respectively. The same volume of sterilized water was administered orally as control experiments.

### 2.4. Administration of SCH23390

SCH23390 hydrochloride (Cayman Chemical, Ann Arbor, MI, USA) was dissolved in saline at 0.05 mg/mL and intraperitoneally administered at a dose of 50 μg/kg 30 min before the oral administration of Matcha tea powder. As control experiments, the same volume of saline was administered intraperitoneally.

### 2.5. Tail Suspension Test

The mice’s depression-like behavior was evaluated by the tail suspension test (TST). The mice were suspended by their tails with tape, and the resultant behavior was recorded by a video camera for 6 min. The behavior of the total duration of immobility was analyzed. For conditioning, the mice were placed in the test room for over 1 h. Matcha tea powder was orally administered 30 min before the beginning of the test.

### 2.6. Open Field Test

The locomotion of the mice was measured for 10 min using video-tracking software (SMART3.0), as previously reported [19]. Matcha tea powder was orally administered 30 min before starting the test.

### 2.7. Immunohistochemistry

Two hours after the Matcha tea powder administration, the mice brains were perfused and fixed, and then, immunohistochemical analysis for c-Fos was performed as described in our previous paper [20]. The number of c-Fos-positive cells in the VTA and PFC regions was counted from 725 × 1092 μm^2^ images. The Core and Shell regions of the NAc were identified according to the brain map, and the number of c-Fos-positive cells within each region was counted (BIOREVO BZ-9000; Keyence, Osaka, Japan). Five sections were collected every 90 μm, and the average value of the number of c-Fos-positive cells per animal was calculated as 1 sample. Rabbit anti-c-Fos antibody (1:2000, CST) and biotinylated anti-rabbit IgG from goat (1:200, Vector Laboratories, Burlingam, CA, USA) were used as the primary and secondary antibody, respectively.

### 2.8. Statistical Analysis

The results are expressed as the means ± SEM. The Prism 6 software (GraphPad Software Inc., San Diego, CA, USA) was used for the statistical analysis. The data were analyzed by Student’s *t*-test in the case of two-group comparisons. Furthermore, the Pearson correlation coefficients were also calculated. In the case of multiple comparisons, statistically significant differences were evaluated with one-way analysis of variance (ANOVA), followed by Dunnett’s or Sidak’s multiple comparison tests. Probability values less than 5% were considered significant.

## 3. Results

### 3.1. Activation of the Dopamine D1 Receptor Contributes to the Antidepressant-like Effects of Matcha Tea Powder in C57BL/6J Mice

**Figure 1:**
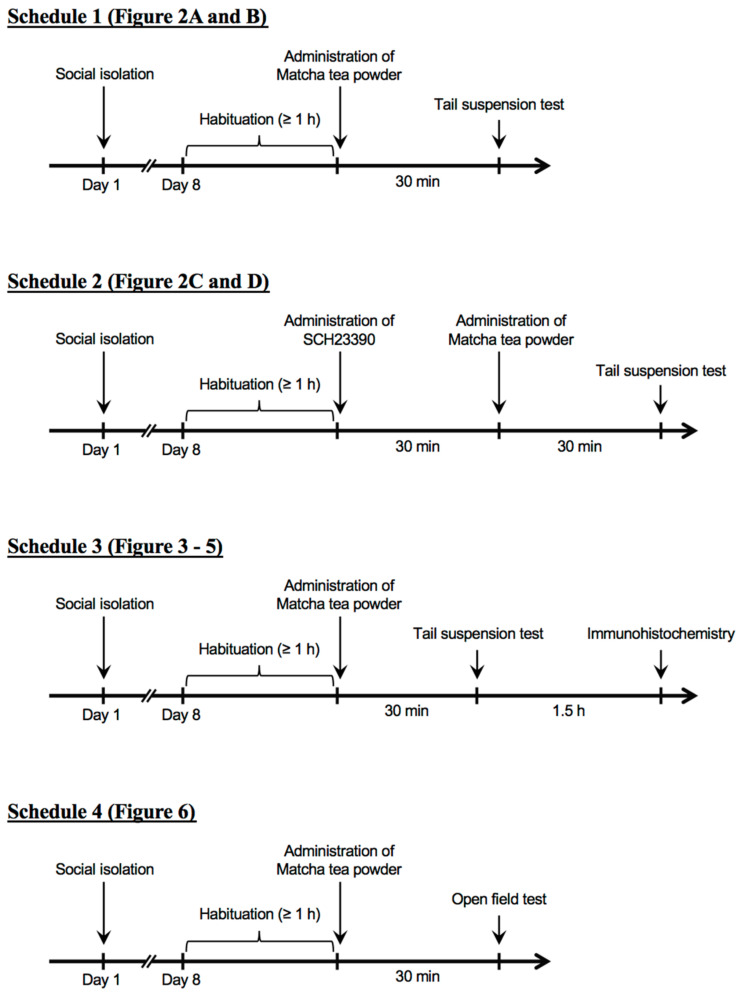
Schematic views of the experimental schedules.

**Figure 2:**
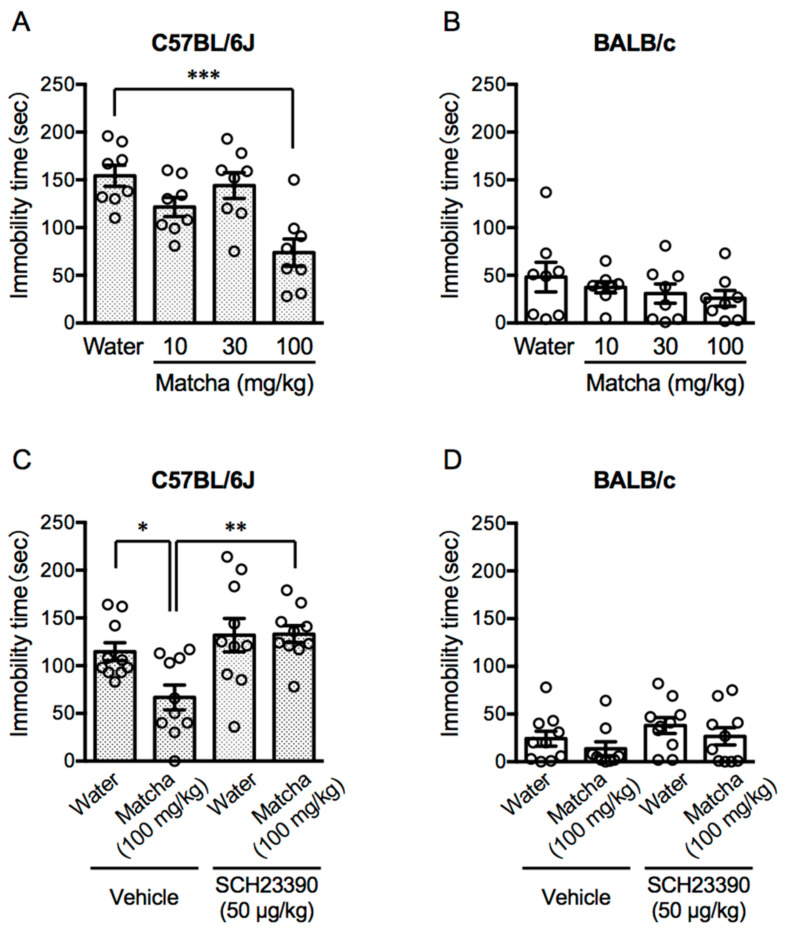
Antidepressant-like effects of Matcha tea powder in stress-vulnerable C57BL/6J mice. The immobility time of C57BL/6J and BALB/c mice in the TST were measured. (**A**,**B**) The TST was performed 30 min after Matcha tea powder administration (10, 30, and 100 mg/kg, p.o.). (**C**,**D**) SCH23390 (50 μg/kg, i.p.) was administered 30 min before the Matcha tea powder administration (100 mg/kg, p.o.). The results are expressed as the means ± SEM. *n* = 8–10 animals. The data were analyzed by one-way ANOVA followed by Dunnett’s multiple comparisons test. * *p* < 0.05, ** *p* < 0.01, *** *p* < 0.001.

Both C57BL/6J and BALB/c mice, which were subjected to social isolation stress, were used in the experiments (Figure 1). The tail suspension test (TST) is the most-common method of evaluating the depression behavior in experimental animals [21]. When mice are suspended in midair with their tails fixed, they behave as if they are resisting the condition. However, the depressed mice spend less time resisting and more time being immobile. In C57BL/6J mice, Matcha tea powder (100 mg/kg, p.o.) shortened the immobility time in the TST (Figure 2A) (*F*_(3, 28)_ = 8.45, *p* = 0.0004; one-way ANOVA). On the other hand, BALB/c mice showed a shorter immobility time after the social isolation stress than the C57BL/6J mice, and oral administration of Matcha tea powder had no effect on the immobility time in TST (Figure 2B) (*F*_(3, 28)_ = 0.8209, *p* = 0.4933; one-way ANOVA).

Next, to investigate the contribution of the dopaminergic systems to the antidepressive property of the Matcha tea powder, we used SCH23390, a dopamine D1 receptor blocker. SCH23390 (50 μg/kg, i.p.) was administered 30 min before the Matcha tea powder administration (100 mg/kg, p.o.). SCH23390 prevented the decrease in the immobility time in C57BL/6J mice, but not in BALB/c mice, receiving Matcha tea powder administration (Figure 2C,D) (C57BL/6J mice; *F*_(3, 36)_ = 6.015, *p* = 0.002, BALB/c mice; *F*_(3, 36)_ = 0.5017, *p* = 0.6835, one-way ANOVA). We also checked that SCH23390 alone had no effect on the depressive levels.

### 3.2. Antidepressive Effect of Matcha Tea Powder Correlates with Neural Activity in the VTA Region in C57BL/6J Mice

**Figure 3:**
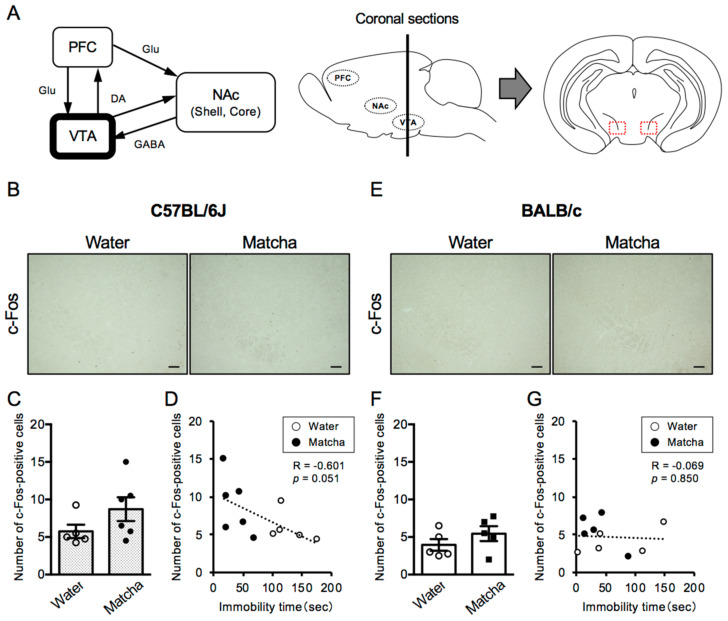
Effects of Matcha tea powder on the neural activity in the VTA region in C57BL/6J and BALB/c mice. (**A**) A schematic view of the PFC-NAc-VTA circuit (dopaminergic system). Dotted lines in red indicate the area of interest for image analysis of the number of c-Fos-positive cells in the VTA region. (**B**,**E**) Representative photographs showing immunohistochemistry on c-Fos in the VTA region 2 h after Matcha tea powder administration (100 mg/kg, p.o.). (**C**,**F**) The quantification of the number of c-Fos-positive cells is shown. The results are expressed as the means ± SEM. The data were analyzed by the unpaired *t*-test. *n* = 5–6 animals. (**D**,**G**) The correlation between immobility time in the TST and the number of c-Fos-positive cells in the VTA region is shown. Scale bars = 100 μm. DA; dopamine, GABA; γ-aminobutyric acid, Glu; glutamate.

To investigate whether the activation state of dopaminergic neural circuits is involved in the antidepressant-like effect of Matcha tea powder, we performed immunohistochemical analysis of c-Fos, a marker of neural activation. In the ventral tegmental area (VTA) region, which is the initiating nucleus of the dopaminergic circuit, the number of c-Fos-positive cells was similar in C57BL/6J and BALB/c mice (*t*_(8)_ = 1.517, *p* = 0.1679, unpaired *t*-test). Moreover, Matcha tea powder (100 mg/kg, p.o.) did not alter the number of c-Fos-positive cells in either C57BL/6J or BALB/c mice (Figure 3) (C57BL/6J mice; *t*_(9)_ = 1.529, *p* = 0.1607, BALB/c mice; *t*_(8)_ = 1.189, *p* = 0.2687, unpaired *t*-test). However, there was a negative correlation between the length of immobility time and the number of c-Fos-positive cells in C57BL/6J mice, but not in BABL/c mice (Figure 3D,G). This suggests that the activation states of the VTA region contribute to the antidepressant-like effect of Matcha tea powder in C57BL/6J mice.

### 3.3. Matcha Tea Powder Increased Neural Activity in the PFC Region in C57BL/6J Mice

**Figure 4:**
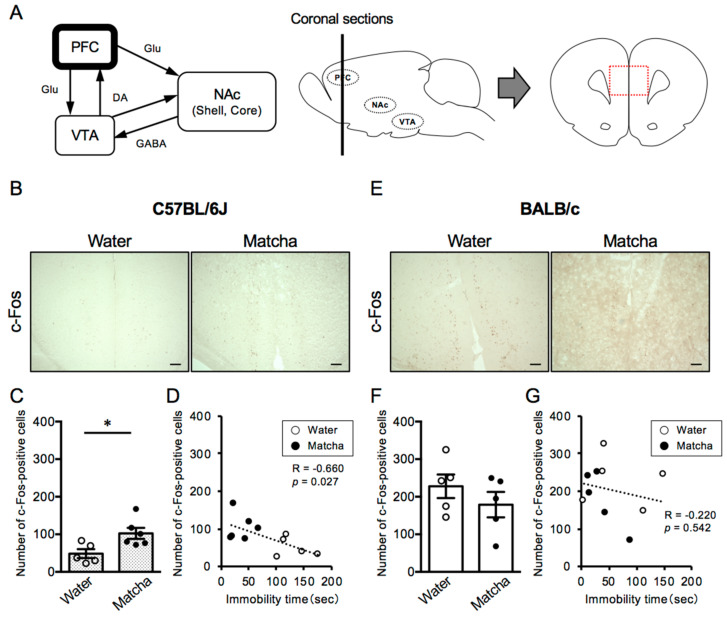
Effects of Matcha tea powder on the neural activity in the PFC region in C57BL/6J and BALB/c mice. (**A**) A schematic view of the PFC-NAc-VTA circuit (dopaminergic system). Dotted lines in red indicate the area of interest for image analysis of the number of c-Fos-positive cells in the PFC region. (**B**,**E**) Representative photographs showing immunohistochemistry on c-Fos in the PFC region 2 h after Matcha tea powder administration (100 mg/kg, p.o.). (**C**,**F**) The quantification of the number of c-Fos-positive cells is shown. The results are expressed as the means ± SEM. *n* = 5–6 animals (**C**) and *n* = 5 animals (**F**), respectively. The data were analyzed by the unpaired *t*-test. * *p* < 0.05. (**D**,**G**) The correlation between immobility time in the TST and the number of c-Fos-positive cells in the PFC region is shown. Scale bars = 100 μm. DA; dopamine, GABA; γ-aminobutyric acid, Glu; glutamate.

Next, we assessed the level of neural activity in the prefrontal cortex (PFC) region, which is associated with the VTA region in the dopaminergic circuit. It is known that neural activity in the PFC region is reduced in patients with depression. As shown in Figure 4, C57BL/6J mice had fewer c-Fos-positive cells in the PFC region than BALB/c mice, indicating lower levels of neural activity (*t*_(8)_ = 5.340, *p* = 0.0007, unpaired *t*-test). Notably, Matcha tea powder (100 mg/kg, p.o.) increased the number of c-Fos-positive cells in C57BL/6J, and there was a strong negative correlation between the length of immobility time and the number of c-Fos-positive cells in the PFC region (Figure 4B–D) (*t*_(9)_ = 2.764, *p* = 0.0220, unpaired *t*-test). On the other hand, in BALB/c mice, Matcha tea powder had no effects on the number of c-Fos-positive cells in the PFC region (Figure 4 E–G) (*t*_(8)_ = 1.060, *p* = 0.3200, unpaired *t*-test). This suggests that the activation states of the PFC region contribute to the depression-like behavior in C57BL/6J and BALB/c mice and are involved in the antidepressant-like effect of Matcha tea powder.

### 3.4. Matcha Tea Powder Increased Neural Activity in the NAc Region in C57BL/6J Mice

**Figure 5:**
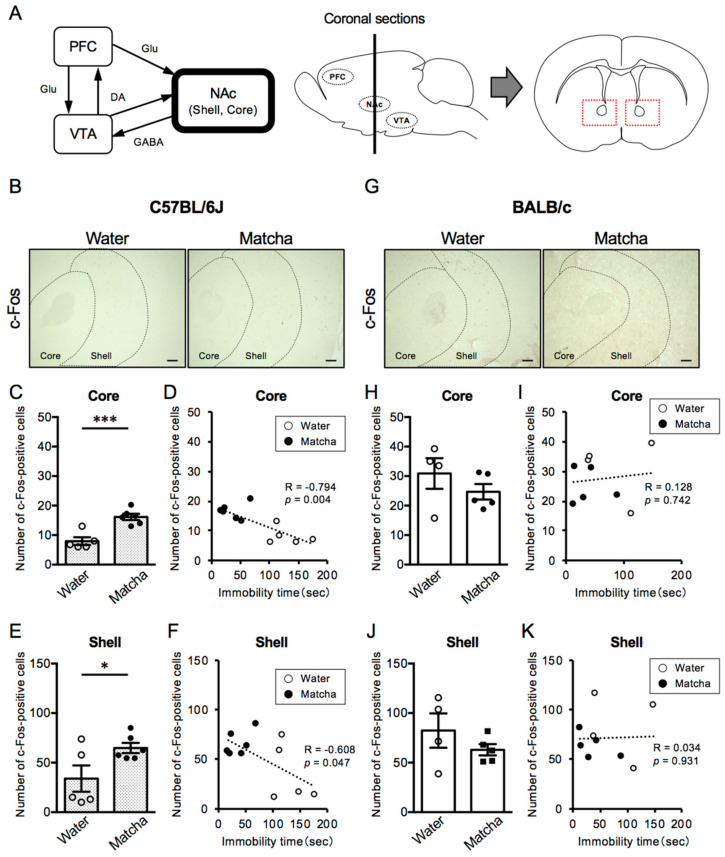
Effects of Matcha tea powder on the neural activity in the NAc region in C57BL/6J and BALB/c mice. (**A**) A schematic view of the PFC-NAc-VTA circuit (dopaminergic system). Dotted lines in red indicate the area of interest for image analysis of the number of c-Fos-positive cells in the NAc region. (**B**,**G**) Representative photographs showing immunohistochemistry on c-Fos in the NAc Core and Shell regions 2 h after Matcha tea powder administration (100 mg/kg, p.o.). (**C**,**E**,**H**,**J**) The quantification of the number of c-Fos-positive cells is shown. The results are expressed as the means ± SEM. *n* = 4–6 animals. The data were analyzed by the unpaired *t*-test. * *p* < 0.05, *** *p* < 0.001. (**D**,**F**,**I**,**K**) The correlation between immobility time in the TST and the number of c-Fos-positive cells in the NAc Core (**D**,**I**) and Shell (**F**,**K**) regions is shown. Scale bars = 100 μm. DA; dopamine, GABA; γ-aminobutyric acid, Glu; glutamate.

In dopaminergic circuits, the nucleus accumbens (NAc), which is functionally classified as Core and Shell regions, is the brain region that directly receives dopaminergic projections from the VTA. The NAc also receives glutamatergic projections from the PFC. In addition, GABAergic neurons also project from the NAc to the VTA and, thus, play an important role in the regulation of emotion and mental state. As shown in Figure 5, C57BL/6J mice, which have a higher degree of depression than BALB/c mice, had fewer c-Fos-positive cells in the NAc Core regions (Core; *p* = 0.0007, Mann–Whitney test, Shell; *t*_(7)_ = 1.670, *p* = 0.1388, unpaired *t*-test). Notably, Matcha tea powder (100 mg/kg, p.o.) increased the number of c-Fos-positive cells in the NAc regions of C57BL/6J mice, but had no effect on BALB/c mice (Figure 5B–K) (C57BL/6J mice; *t*_(9)_ = 4.964, *p* = 0.0008 (Core), *t*_(9)_ = 2.334, *p* = 0.0445 (Shell), BALB/c mice; *t*_(7)_ = 1.135, *p* = 0.2936, *t*_(7)_ = 1.179, *p* = 0.2769, unpaired *t*-test). Furthermore, there was a strong negative correlation between the length of immobility time and the number of c-Fos-positive cells in the NAc Core and Shell regions in C57BL/6J mice. However, BALB/c mice showed a positive correlation between the length of immobility time and the number of c-Fos-positive cells in the NAc Core and Shell regions. These results suggest that the activation states of the NAc regions contribute to the depression-like behavior in C57BL/6J and BALB/c mice and is involved in the antidepressant-like effect of Matcha tea powder.

### 3.5. Matcha Tea Powder Did Not Increase Locomotor Activity in Mice

**Figure 6:**
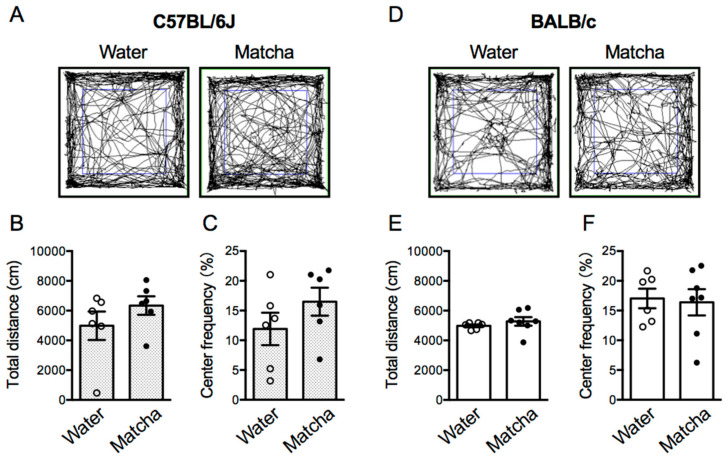
Effects of Matcha tea powder on locomotor activity in the open field test in C57BL/6J and BALB/c mice. (**A**,**D**) Representative photographs showing the trajectory of the mouse in the open field test for 10 min. The total distance traveled in the open field arena (**B**,**E**) and the time spent in the center area (**C**,**F**) were measured. The open field test was performed 30 min after the Matcha tea powder administration (100 mg/kg, p.o.). The results are expressed as the means ± SEM. *n* = 6–7 animals. The data were analyzed by the unpaired *t*-test.

The locomotor activity of mice influences the immobility time in the TST regardless of the depression states, and it is possible that the increase in locomotion causes false-positive results. Here, we performed the open field test 30 min after the Matcha tea powder administration (100 mg/kg, p.o.). The locomotor activities of C57BL/6J and BALB/c mice were nearly identical after social isolation stress, and Matcha tea powder did not alter locomotor activity in either strain (Figure 6) (C57BL/6J mice; *t*_(10)_ = 1.190, *p* = 0.2616, BALB/c mice; *p* = 0.1364, Mann–Whitney test). This suggests that the antidepressant-like effect of Matcha tea powder is not due to increased locomotor activity in socially isolated mice. In addition, we calculated the time spent in the center area and evaluated the anxiety status of the mice. In the open field test, the longer the mice stayed in the center area, the lower the anxiety levels. C57BL/6J mice showed a slightly shorter time spent in the center and a higher anxiety status after social isolation stress compared to BALB/c mice. In C57BL/6J mice, Matcha tea powder tended to increase the time spent in the center area, but the effect was negligible (*t*_(10)_ = 1.270, *p* = 0.2330, unpaired *t*-test). In addition, Matcha tea powder had no effect on the time spent in the center area by BALB/c mice (*t*_(11)_ = 0.2311, *p* = 0.8215, unpaired *t*-test). This suggests that Matcha tea powder ameliorates the depression states and slightly improves the anxiety levels in C57BL/6J mice.

## 4. Discussion

Depression is the most-prevalent mental disorder in the world, and with changing social systems, the number of patients is expected to continue to increase. In the present study, we hypothesized that Matcha tea, a traditional Japanese drink, could improve depression levels, and we evaluated it by the tail suspension test in mice. Generally, Matcha tea is made by dissolving 2–3 g of Matcha tea powder in hot water. We previously reported that Matcha tea powder (50 and 100 mg/kg, p.o.) reduced anxiety states in healthy C57BL/6J mice [19]. Therefore, the dose of Matcha tea powder was set at 10–100 mg/kg in mice to be the same as the standard human dose. Matcha tea is made by mixing whole Matcha tea powder, which contains all the bioactive constituents; therefore, it is expected to have more beneficial effects than green tea, which is made by infusing tea leaves. However, the mechanism that causes individual differences in the physiological effects of Matcha tea powder has not been elucidated. The results we observed provide strong evidence that Matcha improves mood and mental health, depending on the individual’s mental states.

Here, we compared the Matcha tea powder’s effects on mice in different mental states and found that Matcha tea powder exerted its antidepressant-like effect only in C57BL/6J mice under high mental stress due to social isolation. C57BL/6J and BALB/c mice have been reported to have different stress responses [22], and we revealed that the activation states of the PFC and NAc regions in C57BL/6J mice were lower than those of BALB/c mice. These findings are consistent with the fact of the reduced function of the PFC and NAc in the postmortem brains of depressed patients. Animal studies also showed evidence that the neuronal circuits of the PFC-NAc-VTA underlie the development of depression [23]. The PFC controls emotion, mood, and cognition, and depressed patients have a reduced volume of the PFC region and altered levels of GABA and glutamate cycling. Rodent studies also provide evidence of reduced synaptic density and decreased neuronal activity in the PFC region in mice subjected to chronic unpredictable stress (CUS) [24]. The atrophy of the PFC was observed after 1 week of restraint stress (20 to 30 min per day), indicating that the PFC is vulnerable to stress. Thus, even the 1 week of social isolation stress, which was performed in this study, may be enough to reduce the PFC function in C57BL/6J mice. The NAc plays another central role in mood and emotion regulation, and depressive symptoms are also correlated with a reduced NAc volume and reduced NAc responses in patients with major depressive disorder (MDD) [25,26,27]. In rodent studies, chronic stress has been reported to cause hypotrophy of neurons in the NAc, which contributes to disrupted emotion and motivation [28,29]. These reports could explain our results that C57BL/6J mice had worse depression levels than BALB/c mice in response to the stress of 1 week of social isolation.

The involvement of the hypothalamic–pituitary–adrenal (HPA) axis is another possibility of different stress responses between C57BL/6J and BALB/c mice. Stress promotes central and peripheral adaptations via the HPA axis, which can provide feedback to the neurons within the brain through corticosterone release [30]. The increased activity of the HPA axis is thought to be related to altered feedback inhibition by endogenous glucocorticoids, which is mediated by the mineralocorticoid receptor (MR) and glucocorticoid receptor (GR). Notably, impaired sensitivity of the MR and GR, leading to a reduced negative feedback mechanism, is commonly observed in MDD patients [25,31,32]. MR and GR are expressed in the limbic system including the PFC and NAc regions of the brain, which control emotion and motivation, and may be involved in the different stress responses due to social isolation in mice [33,34].

**Figure 7:**
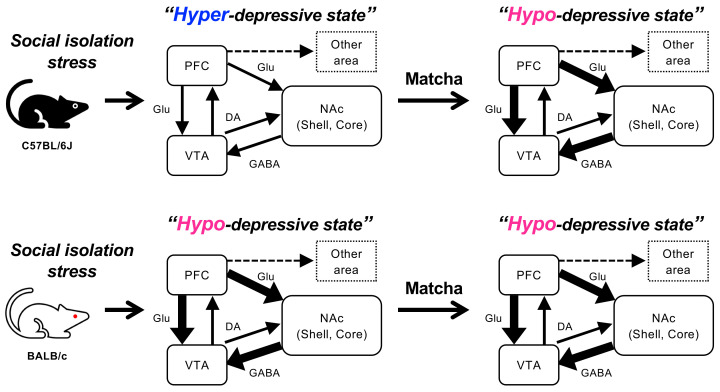
Matcha tea powder exerts antidepressant-like effect by activating the PFC-NAc-VTA circuit (dopaminergic system) in response to the mental states of mice. The depression state differs in strains of mice subjected to social isolation stress. Matcha tea powder exerts an antidepressant-like effect in C57BL/6J mice. Such effects of Matcha tea powder were not observed in BALB/c mice.

In this study, we report for the first time that the antidepressant-like effect of Matcha tea powder is correlated with increased neural activity in the PFC and NAc regions, which are inhibited by the dopamine D1 receptor blockade in C57BL/6J mice. These observations indicate the activation of the dopaminergic systems by Matcha tea powder (Figure 7). The PFC-NAc-VTA circuit is the primary dopaminergic system in the brain, and antidepressants are known to increase neural activity in these regions [35,36,37,38]. Glutamatergic neurons of the PFC form the synapses onto the NAc, which receive dopaminergic inputs from the VTA. Dopaminergic systems originating from the VTA region project axons to the PFC and NAc regions, which control emotional responses by releasing dopamine [39,40]. Furthermore, the activation of dopamine D1 receptors expressed in the PFC and NAc regulate glutamatergic neurons in the PFC and GABAergic neurons in the NAc, respectively, which contributes to enhanced emotional responses [41]. Animal studies have revealed that mice subjected to chronic social defeat stress (CSDS) showed long-term depression in D1-receptor-expressing medium spiny neurons (MSNs), the main projection neurons in the NAc Core, displaying stress susceptibility [42,43,44]. Furthermore, cell-type-specific analysis has revealed the enhanced synaptic strength of D1-MSNs in the NAc Shell in stress-resilient C57BL/6J mice, while the inhibition of these D1-MSNs induced depression-like behavior after CSDS [45,46]. However, contrary to our expectations, the neural activity level in the VTA region was not affected by Matcha tea powder administration. There is a possibility that caffeine, an abundant component of Matcha tea powder, increases dopamine release through the inhibition of adenosine receptors [47]. Furthermore, l-theanine, another major amino acid abundant in Matcha tea powder, increases dopamine and GABA release in the brain [17,48,49]. Caffeine and l-theanine cross the blood–brain barrier within 30 min of administration and have been reported to decrease mental stress and lower anxiety levels in healthy volunteers [50,51]. Another possibility is that Matcha contains agonist-like ingredients directly activating the dopamine D1 receptor. We must also consider that other brain regions are involved in the regulation of the dopaminergic system. For example, the amygdala and hippocampus are other brain regions affecting dopaminergic projections, and dopamine prevents mental failures by suppressing neuronal activity in the amygdala [52,53,54,55]. Further studies are required to measure dopamine levels in the brain and to examine the activation state of the downstream signaling of D1 receptors.

Matcha is expected to enhance motor performance in experimental animals, which may influence the immobility time in the TST regardless of the depression levels. We have previously reported that the locomotor activity of group-housed C57BL/6J mice was increased by the Matcha tea powder administration (100 mg/kg, p.o.) [19]. However, Matcha tea powder did not change the locomotor activity of C57BL/6J and BALB/c mice subjected to social isolation stress. Notably, locomotor activity after social isolation stress was equivalent in C57BL/6J and BALB/c mice, indicating that the antidepressant-like effect of Matcha tea powder is not related to increased locomotor activity.

## 5. Conclusions

By comparing the depressive states of mice with different mental states under social isolation stress, we showed for the first time that Matcha tea powder exerts its antidepressant-like effect only in stress-vulnerable C57BL/6J mice and that the activation states of the PFC-NAc-VTA circuit contribute to such effects. Furthermore, it is noteworthy that the antidepressant-like effect of Matcha tea powder was mediated by the activation of the dopamine D1 receptor. Animal experiments have limitations, and other animal models that reflect the pathophysiology of human depression, such as CSDS, should be considered [56]. Although the mechanisms of dopamine D1 receptor activation in the brain by Matcha tea powder require further investigation, this study contributes to our understanding of the valuable role of Matcha tea powder and provides useful information.

## Data Availability

The data presented in this study are available upon request from the corresponding author.
